# Implementation of Outstanding Electronic Transport in Polar Covalent Boron Nitride Atomic Chains: another Extraordinary Odd-Even Behaviour

**DOI:** 10.1038/srep26389

**Published:** 2016-05-23

**Authors:** Xiaodong Xu, Weiqi Li, Linhua Liu, Jikang Feng, Yongyuan Jiang, Wei Quan Tian

**Affiliations:** 1 Department of Physics, Harbin Institute of Technology, Harbin, 150001, P. R. China; 2 School of Energy Science and Engineering, Harbin Institute of Technology, Harbin, 150001, P. R. China; 3 Institute of Theoretical Chemistry and College of Chemistry, Jilin University, Changchun, 130023, P. R. China; 4 College of Chemistry and Chemical Engineering, Chongqing University, Huxi Campus, Chongqing, 401331, P. R. China

## Abstract

A theoretical investigation of the unique electronic transport properties of the junctions composed of boron nitride atomic chains bridging symmetric graphene electrodes with point-contacts is executed through non-equilibrium Green’s function technique in combination with density functional theory. Compared with carbon atomic chains, the boron nitride atomic chains have an alternative arrangement of polar covalent B-N bonds and different contacts coupling electrodes, showing some unusual properties in functional atomic electronic devices. Remarkably, they have an extraordinary odd-even behavior of conductivity with the length increase. The rectification character and negative differential resistance of nonlinear current-voltage characteristics can be achieved by manipulating the type of contacts between boron nitride atomic chains bridges and electrodes. The junctions with asymmetric contacts have an intrinsic rectification, caused by stronger coupling in the C-N contact than the C-B contact. On the other hand, for symmetric contact junctions, it is confirmed that the transport properties of the junctions primarily depend on the nature of contacts. The junctions with symmetric C-N contacts have higher conductivity than their C-B contacts counterparts. Furthermore, the negative differential resistances of the junctions with only C-N contacts is very conspicuous and can be achieved at lower bias.

The successful isolation of monolayer atomic crystals of graphene and h-BN provides opportunities to shed new insights into physical phenomena and material science^1^,^2^,^3^. Due to their excellent stability and conductivity, the miniaturization of the next-generation devices can be expected to realize in the future. However, those 2D (two-dimensional) crystals are not necessarily the lower limit of spatial confinement. With the top-down method, the amazing linear formation of the monoatomic carbon chains was fabricated using the unconventional approach of a high-energy electron beam irradiating graphene layers^4^,^5^. The free-standing strings consisted of boron and nitrogen atoms were also fabricated with the same approach^6^. Some other physical ways and chemical synthetic approaches can be utilized to fabricate those monatomic chains^7^,^8^,^9^,^10^,^11^,^12^,^13^,^14^,^15^. Due to the ultimate one-atom thinness, their great potential applications could be extended to atomic electronics.

Researches on carbon atomic chains (CACs) have been carried out extensively to investigate its characteristics of unusual electronic transport^16^,^17^,^18^,^19^,^20^,^21^, physical stability^4^,^5^,^22^ and intriguing mechanic properties^23^,^24^, not only at various theoretical levels but also by experiment. More significantly, those one-dimension monoatomic chains can be applied as interconnect wires between 2D materials as two-probe devices. An outstanding character is that the bond type and the transport properties of CACs mainly present even-odd behavior^25^,^26^. The linear geometry of boron nitride atomic chains (BNACs) is similar to its carbon counterpart. For carbon materials and their boron nitride counterparts, the analogy has been confirmed from cage molecules to elongated nanotubes, as well as further to their 2D sheets^1^,^2^,^27^,^28^,^29^,^30^,^31^. Recently, the h-BNC atomic layers structures, hybridized boron nitride and graphene domains, were reported and attracted enormous interest in engineering their bandgaps to improve the varieties of their semiconducting properties^32^,^33^,^34^,^35^,^36^,^37^,^38^. Progresses in 2D materials research pushing toward the growth and isolation of novel 2D structures beyond graphene and h-BN, especially BNC hybrid materials, have stimulated the enthusiasm to explore 1D (one-dimension) monoatomic chain formation composed of B, N, and C elements^39^,^40^,^41^,^42^.

In the present study, first principles simulations are utilized to elucidate the electronic transport properties of BNACs interconnecting symmetric graphene electrodes (GEs) according to the stable connection in hybrid BNC structures. The entire structure is placed in a supercell large enough so that each junction is laterally isolated from its periodic images. To avert the strain effect beyond the axis of the linear chain, the carbon pentagon is symmetrically used to connect the BNACs and this contact form has been investigated previously^43^,^44^. The structures of BNACs have an alternative arrangement of boron and nitrogen atoms and its polar covalent bonds primarily determine the current performance in those interconnect nano-constructions, differing from the even-odd behavior of CACs. The present work is divided into two sections, where the electronic properties and the transport behavior of BNACs bridging GEs with asymmetric and symmetric contacts will be investigated respectively. The BNACs-graphene junctions with asymmetric and symmetric contacts exhibit certain unique transport features, such as rectification character and negative differential resistance, which have great potential applications in quantum transport field.

## Results and Discussion

### Bridging GEs by even BNACs with asymmetric contacts

**Figure 1:**
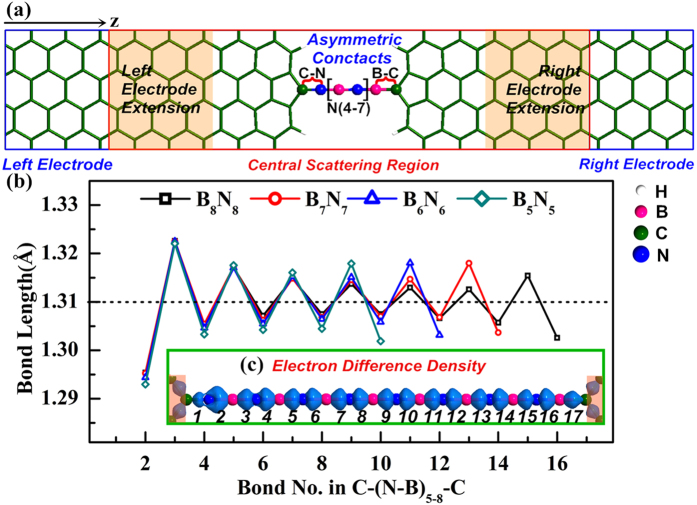
(**a**) Schematic two-probe junctions with asymmetric contacts studied in electronic transport calculation. The junctions are composed of the odd BNACs bridges connected to GEs with C-N contact and C-B contact at two sides. The semi-infinite left and right metallic electrodes are labeled with blue boxes and the red box represents central scattering region containing buffers marked in yellow shadow area as well. z represents the transport direction. (**b**) The bond length alternation with BNACs elongation. (**c**) The electron difference density (B_8_N_8_AC as an example) inset at the bottom of (**b**). The numbers label the bond number. The dots line indicates that the bond lengths in the BNACs fluctuate about 1.31 Å. The atoms are colored as pink for B, blue for N, green for C, and white for H.

For two-probe structures with asymmetric contacts, the electronic transport properties of even atomic BNACs bridges are explored with C-N contact and C-B contact connected to GEs (see Fig. 1). In all asymmetric junctions, the bond lengths of C-N and C-B bond are relaxed to 1.31 Å and 1.46 Å respectively. Character of bond length oscillation similar to CACs with different lengths and terminations was observed^36^,^37^,^38^,^39^. The bond length fluctuating at 1.31 Å is in according with the bond length of the ideal infinite BNACs in a previous theoretical study^6^. The elongation of BNACs makes the bond length oscillation reduces effectively, indicating the enhancement of structural stability. The distribution of electron difference density (as shown in Fig. 1c) reveals the polar nature of the B-N bonds with single-double bond length oscillation. This unique polarized bonds, different from the non-polarized-double bonds of the cumulene (=C = C=) and the non-polarized-triple bonds of the polyyne (-C≡C-), can contribute to the special quantum transport of BNACs nano-constructions. On the other hand, the localization of the electron difference density at contacts reveals that stronger coupling exists in C-N contact than C-B contact, which exerts significant effects on the electrons tunneling between BNACs bridges and GEs.

**Figure 2:**
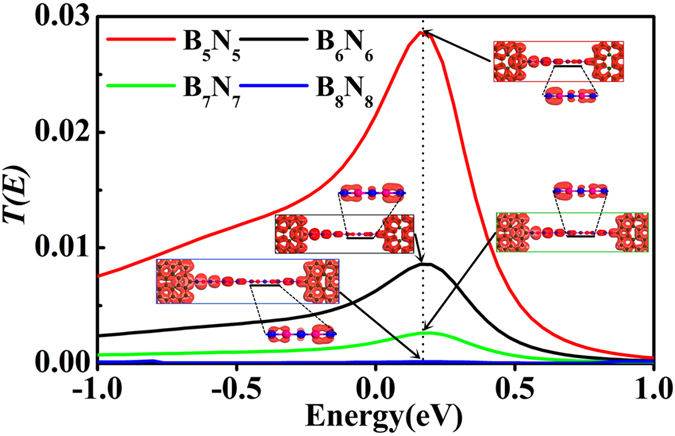
The transmission spectra under equilibrium condition for the asymmetric junctions and the scattering states, local density of states inset in the figure for all asymmetric junctions at *E* = 0.2 eV (the position of resonant tunneling peaks). To make the orbital clear, the side view of the typical orbitals outlined by black line is presented.

Under equilibrium condition, the transmission spectra of the asymmetric contact junctions show a single broad resonant tunneling peak around Fermi level, as illustrated in Fig. 2. Obviously, as the BNACs bridges elongate, the transmission peaks always locate at 0.2 eV, which are contributed by conduction bands. The strengths of those peaks are inversely proportional to the length of bridges. The position and breadth of those peaks signify that the junctions can transmit carriers at low bias and smaller resistance can be achieved when this resonance peak enters bias windows. In order to show the transport pattern clearly, the scattering states of all asymmetric contact junctions at *E* = 0.2 eV are exhibited in Fig. 2. An asymmetric electron distribution is observed at contact regions and chain regions with large π electron density on the two ends of BNACs. At the C-B contacts, there is a single π-orbital between atomic chain and electrode, whereas the π-orbital has anti-bonding character on the C-N contact. In the chain region, the π-orbital locates on an adjacent N-B atom pair as a unit of distribution and there always exists a non-bonding *p*_*y*_-orbital on the N atom close to C-B contact. Thus, from those scattering states patterns, the carriers can be transported feasibly through the C-N contact rather than the C-B contact. The electron redistribution is induced by the graphene at contacts sites, and for different length of BNACs, there is no evident change of the distribution of the electron density on the chain region (see supplementary information). Therefore, couple in the contacts plays a significant role in the conductivity of BNACs.

**Figure 3:**
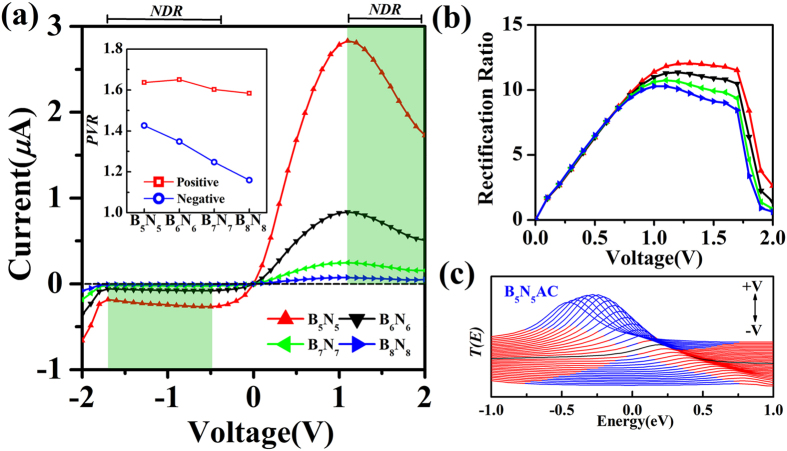
(**a**) The current-voltage characteristics of the asymmetric contact junctions. The NDR regions are denoted by the green shadow area. The inset figure indicates the peak-to-valley rate of NDR with positive bias and negative bias. (**b**) The corresponding rectification ratio as a function of applied bias and the function is defined by *R* = *I*_*Positive*_/*I*_*Negative*_|. (**c**) The evolution of the transmission spectra under various bias. The blue area indicates the expanding bias window and the black solid line represents the transmission spectrum at equilibrium state.

When a bias is applied to the systems, the different coupling in C-N contact and C-B contact primarily dominates the unique rectification phenomenon. Figure 3(a) presents the typical asymmetric current-voltage (*I-V*) characteristics. All those junctions display near linear characteristics in a region between 0.0 V and 0.8 V. The tunneling current of all junctions does not change evidently with the bias increasing from −0.2 V to −1.7 V, where the slope of the *I-V* curves, *dI*/*dV*, is close to zero. Therefore, this region could be taken as “constant region”. To understand the rectification phenomenon, the rectification rate, *R* = |*I*_*Positive*_/*I*_*Negative*_|, was calculated as a function of bias voltages *V*_*b*_ for all asymmetric junctions, where *I*_*Positive/Negative*_ corresponds to the current under positive/negative bias. Several important characteristics can be observed from the rectification ratio as illustrated in Fig. 3(b). For instance, the ratio enhances linearly with the bias increasing from 0.1 V to 0.7 V and has an approximately equal value at the same bias for all asymmetric contact junctions. The correlation between the ratio and voltage could be approximately defined as the formula of *R* = 11.78 *V* + 0.46, where *R/V* represents the rectification ratio/bias voltage respectively. As the bias exceeds 0.7 V, the linear character is broken. The rectification ratio increases to a maximum around 1.1 V for all asymmetric junctions and then reaches a relatively stable stage in the region from 1.0 V to 1.7 V. A sharp decrease, however, occurs at higher bias (exceeds 1.7 V) as the bias window becomes broad enough so that the resonant tunneling peak enters at both polarities.

The variation of transmission spectra *T (E, V*_*b*_) (see Fig. 3(c)) within the expanding bias window at various biases (−1.5 V < *V*_*b*_ < 1.5 V) has significant impact on the rectification phenomenon. Under negative bias, the resonant tunneling peak does not enter the bias window and thus can not contribute to the current significantly. However, under positive bias, large portion and even the whole resonant tunneling peak locates in the bias window so as to cause the evident enhancement in the tunneling current integral. This intrinsic rectification character can significantly make BNACs as rectifier interconnect atomic-wires which is superior to single atomic chains such as metal and carbon atomic chains.

**Figure 4:**
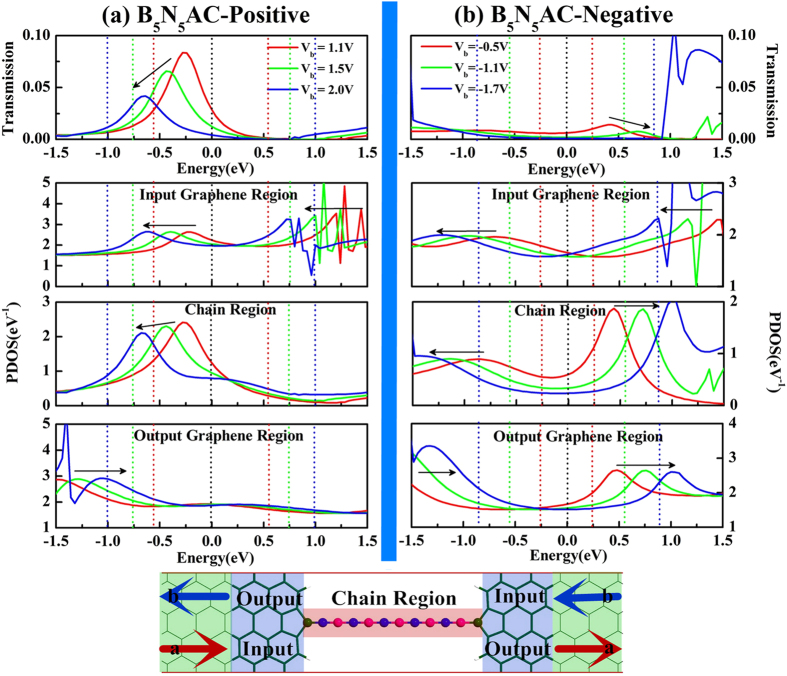
The transmission spectra corresponding to the evolution of scattering-state wave function PDOS (projected density of states) in three scattering regions including input/output graphene region and chain region of the asymmetric junctions under the bias of 1.1 V (red line), 1.5 V (green line), and 2.0 V (blue line) in (**a**) on the left panel and under the bias of −0.5 V (red line), −1.1 V (green line), −1.7 V (blue line) in (**b**) on the right panel. All dash lines indicate the expanding bias windows and the black arrows represent the shift orientation of resonant tunneling peaks in both of transmission spectra and PDOS. The diagrammatic figure is placed at the bottom, where the green shadow, blue shadow and red shadow denotes electrode region, input/output graphene region and chain region, respectively. The red and blue arrows indicate the transport direction of the tunneling current for positive bias and negative bias respectively. B_5_N_5_AC junction is taken as an example and the others have similar patterns.

The other important character of *I-V* curves of asymmetric junctions is the negative differential resistance (NDR) behavior, observed under both positive bias and negative bias (marked with green shadow region in Fig. 3(a)). Generally, resonance tunneling occurs when carrier transports in a well-matched energy eigenstate in junctions. However, not only the chemical potential of electrodes but also the position of the resonant levels can be shifted by applied bias. Since the electronic partial density of states (PDOS) gives information of individual orbital of an atom or a group of atoms contributing to the eigenchannels of junctions^16^,^45^,^46^,^47^,^48^,^49^, the underlying mechanism of NDR can be expounded by the shift of PDOS derived from the central scattering region which is divided into input graphene region, chain region, and output graphene region. Before the bias moves into NDR region (see Fig. 3(a)), part of the transmission peak enters the bias window gradually, resulting in an increase in current. Meanwhile, the eigenstate channels are well-matched gradually. With the bias continuously increasing, the well-matched states are modified and then the mismatched states appears. In Fig. 4(a), although the resonant tunneling peak moves into the expanding bias window at the bias of 1.1 V, 1.5 V, and 2.0 V, the strength is gradually reduced by the applied bias. In combination with the PDOS in the three divided regions of the central scattering region, both of the position and shift direction of the transmission peak line up well with the prominent peak in both chain region and input graphene region. In addition, the reduction of transmission peak strength is also reflected by the variation of the PDOS peak in the chain region. As the bias increases, the PDOS peaks of the chain region and the input graphene region synchronously move to valence band, while the PDOS in output graphene region moves to conduction band. Thus, the miss-matched state appears between chain region and output graphene region until the applied bias drives the PDOS peak (marked with arrow in output graphene region of Fig. 4(a)) of valence band to match the resonant tunneling states of the chain region. As a result, a net drop in current from 1.1 V to 2.0 V is caused by the mismatch of the PDOS in the divided regions, resulting in a positive NDR in *I-V* characteristics.

For the negative NDR, a similar explanation holds. Although the evolution of transmission spectra in constant region is un-conspicuous, there is still a weak NDR. From peak to valley, the transmission spectra of the selected biases of −0.5 V, −1.1 V, −1.7 V are presented (see Fig. 4(b)) and there presents a weak peak within the expanding bias windows of which shift direction is inverse to that of positive bias. There are two PDOS peaks appearing in chain region but in opposite shift direction. Evidently, both of the peaks are responsible for the transmission spectra within the expanding bias windows. As a consequence, the negative NDR is caused by the PDOS of chain region above Femi level mismatching the PDOS of input graphene region and below Fermi level mismatching the PDOS of output graphene region (see the evolution of the PDOS marked with arrow in Fig. 4(b)). Furthermore, both positive NDR and negative NDR are characterized by the so-called peak-to-valley rate (PVR), which is the rate between the maximal (peak) and the minimal (valley) current. With the BNACs elongation, the PVR is plotted and inset in Fig. 3(a), in which the positive PVR remains relatively constant at 1.62 while negative PVR decreases gradually.

### Bridging GEs by odd BNACs with symmetric contacts

**Figure 5:**
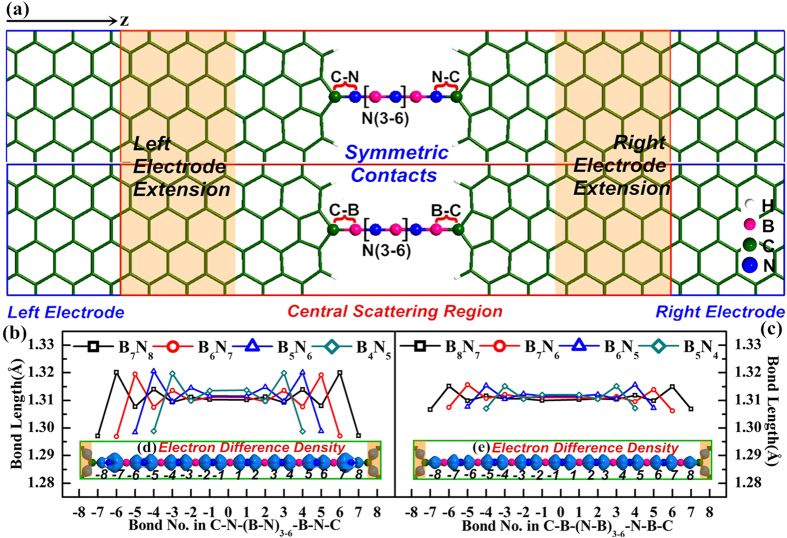
(**a**) Sketch of symmetric contact junctions with only C-N contacts (top panel) and only C-B contacts(bottom panel). (**b**,**c**) The oscillation of bond length with chain elongation. (**d**,**e**) The electron difference density of B_7_N_8_AC and B_8_N_7_AC (taken as examples, and the others are similar). The numbers label the bond number.

Two types of symmetric junctions (odd BNACs bridging GEs with only C-N contacts or C-B contacts) are presented in Fig. 5(a). Similar to the asymmetric junctions, the bond length of C-N contact and C-B contact is 1.31 Å and 1.46 Å for all symmetric junctions, respectively. Except the bond of contacts, the bond lengths oscillate in the range from 1.295 Å to 1.320 Å for the symmetric C-N contact junctions (presented in Fig. 5(b)) and from 1.305 Å to 1.315 Å for the symmetric C-B contact junctions (presented in Fig. 5(c)). The symmetric electron difference density distribution and polar covalent B-N bonds can be observed in the BNACs (see the Fig. 5(d,e)). The localized electron density on C-N contact and C-B contact indicates that strong coupling is realized by the C-N contact and electron transports through the C-N contact more feasibly compared with the C-B contacts.

**Figure 6:**
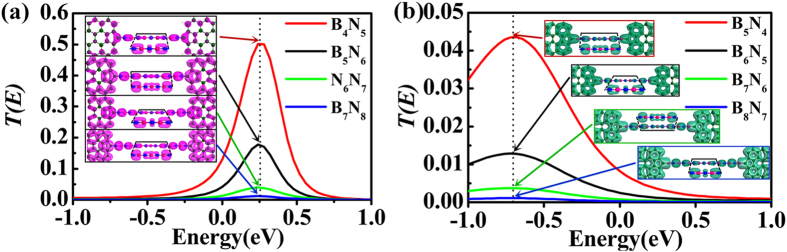
(**a**) The transmission spectra of the symmetric C-N contact junctions under zero bias and the scattering states with LDOS at 0.25 eV (the position of transmission peak). (**b**) The transmission spectra of the symmetric C-B contact junctions under zero bias and the scattering states with LDOS at −0.7 eV (the position of the transmission peak). To show the orbital clearly, the side view of the typical orbitals outlined by black line is presented.

The transmission spectra of two types of symmetric junctions under zero bias are presented in Fig. 6. The resonant tunneling peak of the symmetric C-N contact junctions locating above Fermi level (as shown in Fig. 6(a)) has higher conductivity than the symmetric C-B junctions with resonant tunneling peak below Fermi level (as shown in Fig. 6(b)). The strength of the transmission peak of junctions with only C-N contacts is one order of magnitudes larger than the C-B contact junctions with the same size. Additionally, the simulated scattering states for both of the symmetric junctions with BNACs elongation are inset in Fig. 6(a,b) respectively. Coinciding with the orbital distribution in asymmetric junctions, there is also anti-bonding π-orbital localized on the C-N contact and a π-orbital on the C-B contact. The distribution of π-orbitals on chains has a unique variation (see Fig. 6(a,b)). If the symmetric center is N atom (such as B_4_N_5_AC, B_6_N_7_AC, B_6_N_5_AC and B_8_N_7_AC), a non-bonding *p*_*y*_-orbital locates on the symmetric center N atom. However, if the symmetric center is B atom, an unusual π-orbital delocalizes on the central three atoms containing symmetric center B atom and the neighboring two N atoms (such as B_5_N_6_AC, B_7_N_8_AC, B_5_N_4_AC and B_7_N_6_AC). According to the orbital distribution, the transport properties of graphene-BNACs junctions can be tuned by manipulating the contacts to achieve different conductivities.

**Figure 7:**
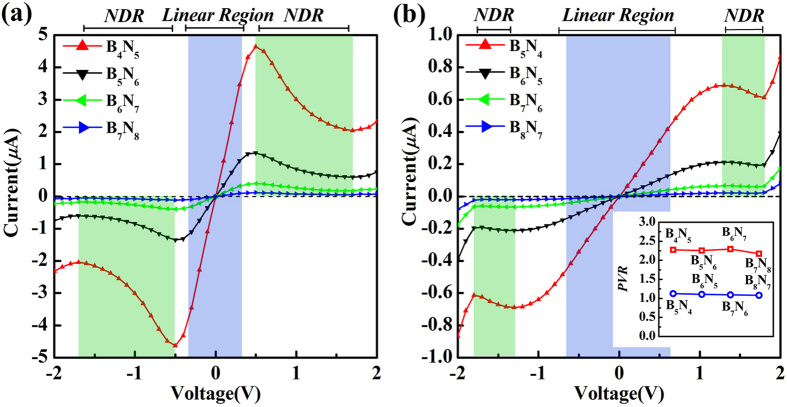
The symmetric current-voltage characteristics of the two types of symmetric junctions, (**a**) for the symmetric C-N contact junctions and (**b**) for the symmetric C-B contact junctions. The shadow regions of blue and green show linear region and NDR region. The peak-to-valley rate is plotted for all symmetric junctions and inset in (**b**).

Contrary to the asymmetric junctions, there presents symmetric *I-V* characteristics for the symmetric junctions (see Fig. 7(a,b)). The NDR behavior is observed for both types of symmetric junctions as well, highlighted by the green shadow region. Especially to deserve to be emphasized, the NDR of the symmetric C-N contact junctions can be generated at low applied bias of 0.5 V. However, an un-conspicuous NDR appears in symmetric C-B contact junctions. Additionally, the linear region of *I-V* curves for the junctions with C-B contacts has wider bias range as marked with blue shadow in Fig. 7(b). The peak-to-valley rate of NDR also has been calculated and inset in Fig. 7(b), where the PVR keeps stable with BNACs elongation for both types of symmetric junctions, and the rate of the junctions with only C-N contacts is twice larger than their C-B contacts counterparts.

**Figure 8:**
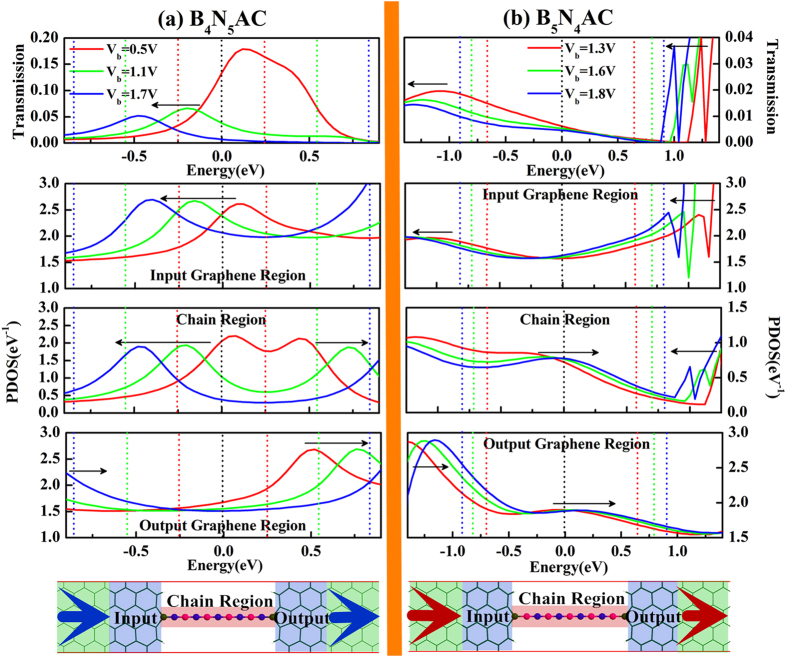
The transmission spectra corresponding to the evolution of scattering-state wave function PDOS (projected density of states) in three divided regions of 0.5 V (red line), 1.1 V (green line), and 1.7 V (blue line) in (**a**) on the left panel for the symmetric C-N contacts junction and under the bias of 1.3 V (red line), 1.6 V (green line), 1.8 V (blue line) in (**b**) on the right panel for the symmetric C-B contact junction. All dash lines indicate the bias windows and the black arrows represent the shift orientation of resonant tunneling peaks in both of transmission spectra and PDOS. The diagrammatic figure is placed at the bottom, where the green shadow, blue shadow and red shadow denotes electrode region, input/output graphene region and chain region, respectively. The arrows in GEs indicate the transport direction of the tunneling current for positive bias B_4_N_5_AC junction and B_5_N_4_AC junction are taken as examples and the others have similar patterns.

In Fig. 8(a), B_4_N_5_AC taken as an example, the applied bias shifts the transmission peak to valence bands and reduces the strength of the transmission peak simultaneously, resulting in substantial decrease in current. In the expanding bias window, the transmission peak is contributed by the prominent PDOS peak in the input graphene region and chain region. With applied bias increases, the PDOS peaks in the input graphene region and output graphene region move to valence band and conduction band, respectively. However, for the chain region, one of the splitting peaks moves to valence band which primarily contributes to the transmission peak, while the other shift to conduction band beyond the bias window. The mismatched resonant tunneling states occur between the PDOS of the chain region and output graphene region below the Fermi level, resulting in NDR character. In Fig. 8(b), B_4_N_5_AC taken as an example, the transmission reduction still is attributed to the variation of PDOS in chain region. The mismatch is slightly caused by the opposite shift of the PDOS between chain region and output graphene region in the narrow bias region of [1.3 V, 1.8 V]. When bias exceeds 1.8 V, the robust transmission peak (marked with arrow on the right side of transmission spectra in Fig. 8(b)) moves into the bias window resulting in a sharp enhancement in tunneling current eventually. As a result, there is only a narrow and weak NDR in symmetric C-B contact junctions.

Furthermore, the effect of the length of BNACs on the conductivity and charge redistribution of the chains spanning between graphene layers has been investigated in detail (see supplementary information). The decrease of conductivity eventually to insulator is clearly revealed as shown in Fig. S1 and the charge distribution has no significant change as the chain elongates as shown in Fig. S2. For the three type of junctions, their different conductivity can be ascribed to the electron redistribution at the contacts induced by graphene electrodes. Significantly, there presents an extraordinary odd-even behavior of conductivity differing from CACs.

## Conclusion

The point-contacts influence on the electronic transport properties of BNACs-graphene junctions has been investigated by first principles calculations. Differing from CACs, BNACs exhibits an interesting odd-even behavior of conductivity. A certain nonlinear *I-V* characteristics, such as rectification character and negative differential resistance, can be achieved and manipulated by the contacts between BNACs bridges and GEs. The intrinsic rectification is caused by the electron redistribution in even BNACs and the different coupling in C-N contact and C-B contact. The point-contacts influence is further demonstrated by the BNACs connected to GEs through symmetric contacts, for instance, the junctions with C-N contacts have higher conductance than with C-B contacts. Furthermore, for the three typical junctions, the negative differential resistances, caused by large applied bias which leads to the mismatch of the resonant tunneling states in the three parts of the central scattering region, are also dominated by different contacts. For the symmetric C-N contact junctions, the large PVR can be achieved at lower applied bias. The significance of the point-contacts influence on the transport properties of the BNACs-graphene junctions warrants those nano-structures for applications in atomic electronic devices.

### Computational Details

The electron transport properties of all two-probe junctions were explored by utilizing the non-equilibrium Green’s function approach in combination with density functional theory (NEGF + DFT). The geometry optimization for each junction is performed firstly with density functional theory (DFT) as implemented in the Vienna ab initio simulation package (VASP)^50^,^51^ and then by the Atomistix Toolkits software package (ATK)^52^,^53^ until the absolute forces on each atom is less than 0.02 eV/Å. The valence electronic orbitals of the junctions were described with double zeta polarized basis set. The GGA-PBE (the generalized gradient approximation with the Perdew-Burke-Ernzerhof parametrization) was utilized as the exchange and correlation functional for all calculation of electron-electron interaction^54^. The transport calculations were obtained using ATK package, which employs the approach of NEGF + DFT, a cutoff energy of 150 Ry was enough to guarantee computational precision and the Brillouin zone was sampled as Monkhost-Pack grid using 2 × 2 × 100 *κ*-points. Note that all atoms were described self-consistently at the same level of theory for not only the central region but also the electrodes and this approach has been thoroughly expounded in the previous works^55^,^56^.

The transmission coefficient *T (E*) for electrons of energy *E* (passing from the source to drain) is calculated via the relation:


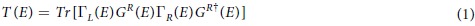


where Γ_*L,R*_(*E, V*_*b*_) = *i* [Σ_*L,R*_(*E*) − Σ_*L,R*_^†^(*E*)] describes the level broadening because of the coupling between left and right electrodes and the central region. Σ_*L, R*_ (*E*) are the retarded self-energies associated with this coupling, and *G*^*R*^ = (*ES − H* − Σ_*L*_ − Σ_*R*_)^−1^ is the retarded Green’s function, where *H* is the Hamiltonian and *S* is overlap matrix. In addition, the current through the junctions under bias *V*_*b*_ is defined by the Landauer-Büttiker formula^57^,^58^:


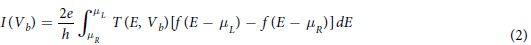


where *f*(*E*) = [1 + exp((*E − E*_*F*_)*/k*_*B*_*T*)]^−1^ is the Fermi-Dirac distribution function, *T* is temperature, and *k*_*B*_ is Boltzmann’s constant. *μ*_*L*_ = *E*_*F*_ + *eV/2* and *μ*_*R*_ = *E*_*F*_ − *eV/2* represent the chemical potential of left and right electrode respectively, [*μ*_*L*_*, μ*_*R*_] denotes the bias window.

## Additional Information

**How to cite this article**: Xu, X. *et al*. Implementation of Outstanding Electronic Transport in Polar Covalent Boron Nitride Atomic Chains: another Extraordinary Odd-Even Behaviour. *Sci. Rep.* **6**, 26389; doi: 10.1038/srep26389 (2016).

## Supplementary Material

Supplementary Information
