# Supplementary material for: Implementation of Outstanding Electronic Transport in Polar Covalent Boron Nitride Atomic Chains: another Extraordinary Odd-Even Behaviour
Source: Sci Rep. 2016 May 23;6:26389. doi: 10.1038/srep26389 (PMC4876473; doi:10.1038/srep26389)
Supplement: Supplementary Information [file srep26389-s1.doc]

**Supplementary Information**

**Implementation of Outstanding Electronic Transport in Polar Covalent Boron Nitride Atomic Chains: another Extraordinary Odd-Even Behaviour**

**Xiaodong Xu^1^, Weiqi Li^1^,*, Linhua Liu^1, 2^, Jikang Feng^3^, Yongyuan Jiang^1^,*, and Wei Quan Tian^4^,***

^1^Department of Physics, Harbin Institute of Technology, Harbin, 150001, P. R. China

^2^School of Energy Science and Engineering, Harbin Institute of Technology, Harbin, 150001, P. R. China

^3^Institute of Theoretical Chemistry and College of Chemistry, Jilin University, Changchun, 130023, P. R. China

^4^College of Chemistry and Chemical Engineering, Chongqing University, Huxi Campus, Chongqing 401331, P. R. China

^*^Correspondence and requests for materials should be addressed to W.L. (email: tccliweiqi@hit.edu.cn) or W.Q. T. (email: [tianwq@cqu.edu.cn](mailto:tianwq@cqu.edu.cn) )


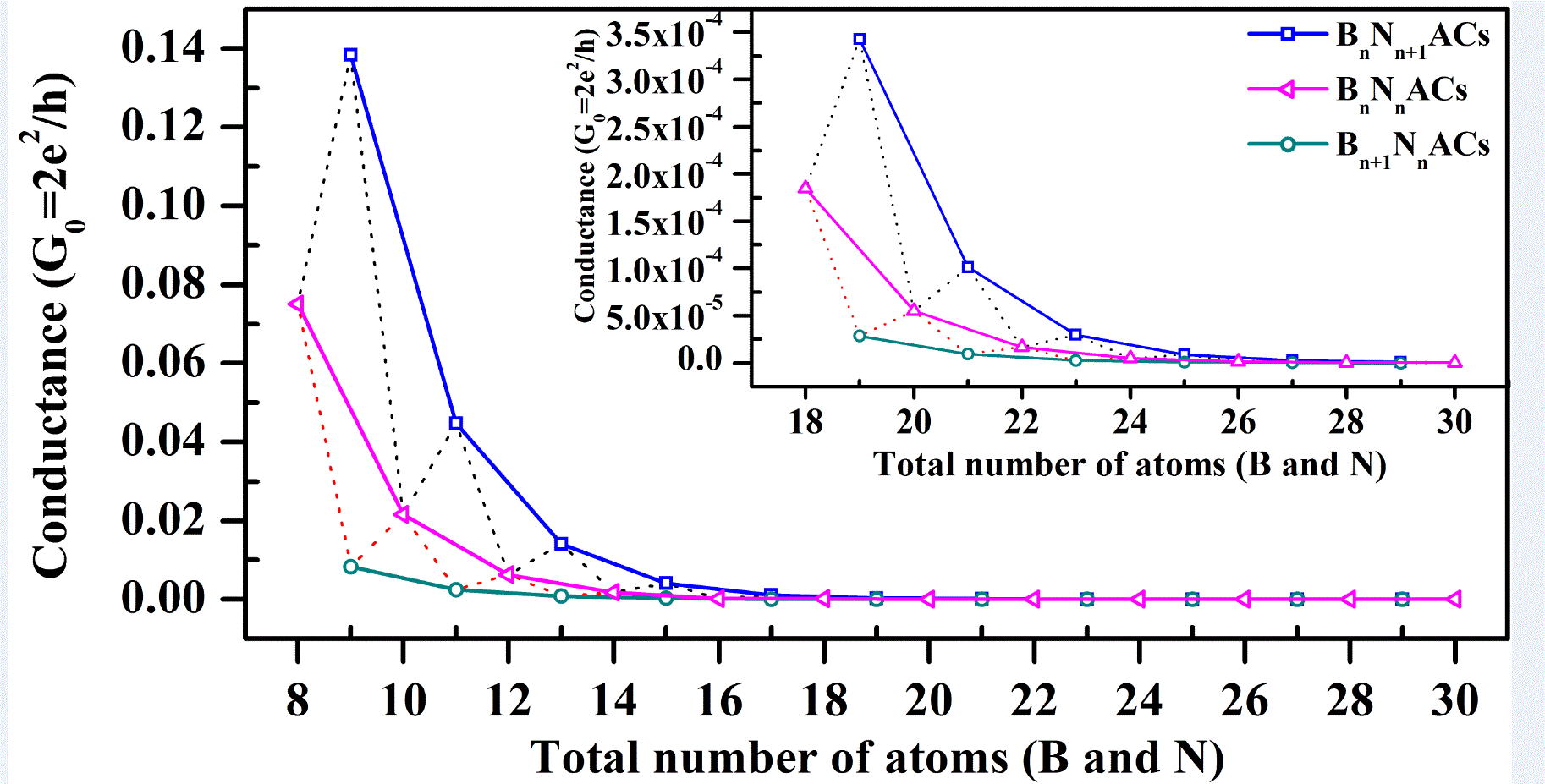


Figure S1. The evolution of conductivity of BNACs-GEs junctions as a function of chain length

Due to the different coupling between C-B and C-N, the electronic transport of the BNACs bridging graphene electrodes shows an extraordinary odd-even behavior of conductivity differing from carbon atomic chains. However, with the BNACs elongation, the conductivity of the junctions decreases rapidly, namely, infinitely long BN chains would be insulating. To present the evolution of the odd-even behavior of conductivity with the length increase clearly, we plot the conductivity at Fermi level as a function of the total number of atoms (including B and N atoms ), as shown in Figure S1. From figure S1, we confirm that the conductivity decrease rapidly with the number of atoms increasing and three kinds of atomic arrangement in BNACs determine the types of contacts between BNACs and graphene layers and further influence the electrical conductivity of the junctions. The junctions with symmetric C-N contacts perform better conductivity than others, while the junctions with symmetric C-B contacts is always insulter. For three types of junctions, the conductivity shows an exponential damping with atomicity increasing. More significantly, there are two patterns of odd-even behavior. One is that the conductivity jump between the B_n_N_n+1_ACs(the blue line) and B_n_N_n_ACs (the pink line) performing a zigzag oscillation ( the black dot line). Another is the zigzag oscillation (the red dot line) jumping between B_n+1_N_n_ACs(the green line) and B_n_N_n_ACs(the pink line). Between this two patterns shows a π phase difference. The extraordinary odd-even behavior and the decrease conductivity would be always present with the length increase.


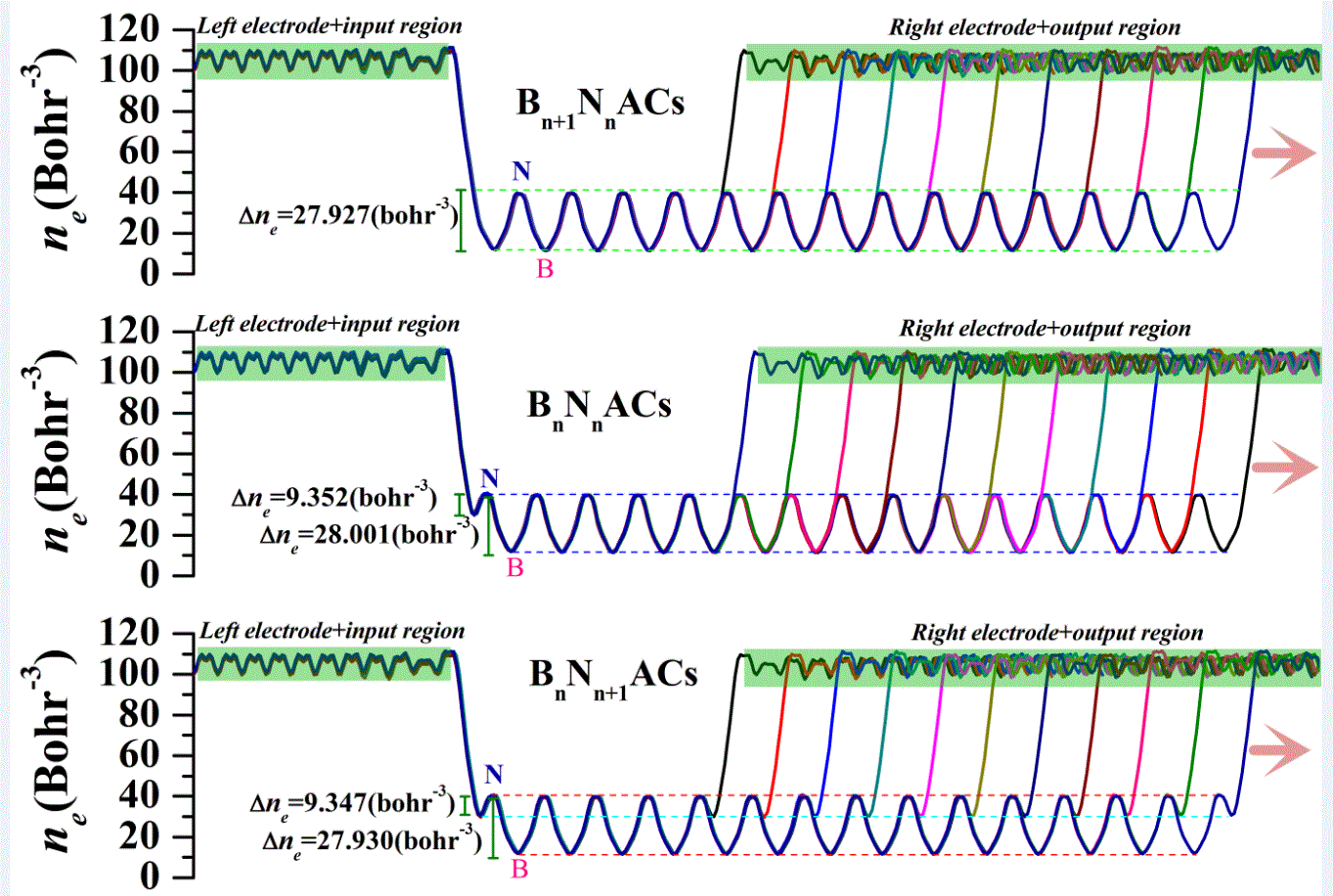


Figure S2. The evolution of electron density distribution with the chain length increase for the three types of BNACs junctions.

On the other hand, we also investigate the variation of the electron density with the length increase, as shown Figure S2. The Figure presents the distribution of the electron density along z-direction (transport direction) and perpendicular to the x-y planar. With the BNACs elongation, the distribution of the electron density on the chains region shows a sinusoidal function. Simultaneously, the period and amplitude are not altered by the length of BNACs. The difference between peak and valley in chain region is 27.927, 28.001, 27.930 for B_n+1_N_n_ACs, B_n_N_n_ACs and B_n_N_n+1_ACs, respectively. Near the contacts region, however, the distribution of the electron density presents larger different between C-N contact and C-B contact. At C-N contact, the distribution of the electron density shows a small dip where the difference relative to peak of sinusoidal function is 9.427 and 9.396 for B_n_N_n_ACs and B_n_N_n+1_ACs respectively, while the electron density between C and B atom always keeps degenerative. As a result, the electron redistribution is induced by the graphene at contacts sites, and for different length of BNACs, there is no evident change of the distribution of the electron density on the chain region (except contacts sites).
